# Synaptic Activity and Muscle Contraction Increases PDK1 and PKCβI Phosphorylation in the Presynaptic Membrane of the Neuromuscular Junction

**DOI:** 10.3389/fnmol.2017.00270

**Published:** 2017-08-25

**Authors:** Erica Hurtado, Víctor Cilleros, Laia Just, Anna Simó, Laura Nadal, Marta Tomàs, Neus Garcia, Maria A. Lanuza, Josep Tomàs

**Affiliations:** Unitat d’Histologia i Neurobiologia (UHNEUROB), Facultat de Medicina i Ciències de la Salut, Universitat Rovira i Virgili Reus, Spain

**Keywords:** PDK1, cPKCβI, phosphorylation, neuromuscular junction, PKC, muscle contraction

## Abstract

Conventional protein kinase C βI (cPKCβI) is a conventional protein kinase C (PKC) isoform directly involved in the regulation of neurotransmitter release in the neuromuscular junction (NMJ). It is located exclusively at the nerve terminal and both synaptic activity and muscle contraction modulate its protein levels and phosphorylation. cPKCβI molecular maturation includes a series of phosphorylation steps, the first of which is mediated by phosphoinositide-dependent kinase 1 (PDK1). Here, we sought to localize PDK1 in the NMJ and investigate the hypothesis that synaptic activity and muscle contraction regulate in parallel PDK1 and cPKCβI phosphorylation in the membrane fraction. To differentiate the presynaptic and postsynaptic activities, we abolished muscle contraction with μ-conotoxin GIIIB (μ-CgTx-GIIIB) in some experiments before stimulation of the phrenic nerve (1 Hz, 30 min). Then, we analyzed total and membrane/cytosol fractions of skeletal muscle by Western blotting. Results showed that PDK1 is located exclusively in the nerve terminal of the NMJ. After nerve stimulation with and without coincident muscle contraction, total PDK1 and phosphorylated PDK1 (pPDK1) protein levels remained unaltered. However, synaptic activity specifically enhanced phosphorylation of PDK1 in the membrane, an important subcellular location for PDK1 function. This increase in pPDK1 coincides with a significant increase in the phosphorylation of its substrate cPKCβI also in the membrane fraction. Moreover, muscle contraction maintains PDK1 and pPDK1 but increases cPKCβI protein levels and its phosphorylation. Thus, even though PDK1 activity is maintained, pcPKCβI levels increase in concordance with total cPKCβI. Together, these results indicate that neuromuscular activity could induce the membrane targeting of pPDK1 in the nerve terminal of the NMJ to promote the phosphorylation of the cPKCβI, which is involved in ACh release.

## Introduction

Protein kinase C (PKC) is a common signaling node of many cellular processes, being a crucial regulator of neuronal excitability, neurotransmitter release and synaptic transmission in the nervous system (Dempsey et al., 2000; Lanuza et al., 2014; Tomàs et al., 2014). Several PKC isoforms are expressed and differently regulated in the skeletal muscle and, particularly, at the neuromuscular junction (NMJ; Hilgenberg and Miles, 1995; Lanuza et al., 2000; Perkins et al., 2001; Li et al., 2004; Besalduch et al., 2010, 2013; Obis et al., 2015a,b). Specifically, the conventional PKC βI (cPKCβI) has been involved in the regulation of diverse cellular functions including neurotransmission (Hurtado et al., 2017). It is located exclusively at the nerve terminals of NMJ and muscle contraction retrogradely enhances its levels through the brain-derived neurotrophic factor (BDNF)/tropomyosin receptor kinase B (TrkB) signaling (Hurtado et al., 2017).

PKC subcellular location is closely related with its activity. Different evidence show that PKC undergoes a process of maturation before catalytic competence (Parekh et al., 2000; Newton, 2003). In order to mature, PKC undergo a series of three phosphorylations, the first of which is mediated by phosphoinositide-dependent kinase 1 (PDK1). Membrane location confers to PKC a permissive change that enables PDK1 to access and phosphorylate its activation loop. The mature cPKCs, now “primed” for activation, are released into the cytosol and kept in an inactive conformation (Oancea and Meyer, 1998; Violin et al., 2003; Griner and Kazanietz, 2007). In the presence of intracellular calcium, diacylglycerol (DAG) and phosphatidylserine, cPKCs are tethered to the membrane ready for substrate binding, phosphorylation and the activation of downstream signaling effectors (Colón-González and Kazanietz, 2006). After their activation, PKC is downregulated through a poorly understood mechanism. In particular, the short half-life of DAG could be important for cPKC signaling termination. However, a ubiquitin–proteasome-dependent pathway for PKC isoforms has also been described (Lee et al., 1996; Lu et al., 1998; Leontieva and Black, 2004). Recent findings show that PKCs might also be present in non/hypophosphorylated forms being their phosphorylation inducible after cellular stimulation (Zhou et al., 2003; Wang et al., 2007; Osto et al., 2008; Freeley et al., 2011). Consistent with these authors, we recently found that synaptic activity enhances the phosphorylation of cPKCβI (Hurtado et al., 2017). As stated above, cPKCβI has a key role in the regulation of neurotransmission in the presynaptic component of the NMJ. Therefore, the mechanisms involved in maturation and activation of cPKCβI must be identified if the physiological functions of this isoform are to be better understood.

The discovery of PDK1 as the upstream kinase for PKCs represented an important step towards understanding PKC regulation. PDK1 is a serine (Ser)/threonine (Thr) kinase which needs to be targeted to the plasma membrane to interact with and phosphorylate its substrates such as PKC (Chou et al., 1998; Dutil et al., 1998; Le Good et al., 1998; Balendran et al., 2000). Although the action of PDK1 on PKC signaling has been extensively studied, how PDK1 activity is regulated is still controversial and whether PDK1 is modulated by synaptic activity in the NMJ remains unknown.

In the current study, we localized PDK1 at the NMJ and we investigated the hypothesis that synaptic activity and muscle contraction regulates PDK1 and its substrate cPKCβI phosphorylation in the membrane fraction.

## Materials and Methods

### Animals

“Diaphragm and levator auris longus (LAL) muscles of Sprague-Dawley rats (45–50 days; Criffa, Barcelona, Spain; RRID: RGD_5508397) were used to perform stimulation experiments, Western Blot and Immunohistochemistry. The animals were cared for in accordance with the guidelines of the European Community Council Directive for the humane treatment of laboratory animals. At least five independent animals (*n* > 5) were used to evaluate the following techniques” (Hurtado et al., 2017).

### Antibodies

Primary antibodies used for Western blotting were mouse monoclonal anti-PDK1 (Cat# sc-17765 RRID: AB_626657), rabbit anti-PKCβI (Cat# sc-209 RRID: AB_2168968) and goat anti-glyceraldehyde 3-phosphate dehydrogenase (GAPDH); (Cat# sc-20358 RRID: AB_641101) polyclonal antibodies, purchased from Santa Cruz Biotechnology. Mouse monoclonal anti-Na/K-ATPase antibody was purchased from Developmental Studies Hybridoma Bank. Rabbit anti-pPKCβI (Thr642; Cat# ab75657 RRID: AB_1310586) polyclonal antibody was purchased from Abcam. Rabbit anti-phosphorylated PDK1 (pPDK1; Ser241; Cat# 3061S RRID: AB_2161919) polyclonal antibody was purchased from Cell Signaling Technology.

The secondary antibodies used were donkey anti-rabbit conjugated to horseradish peroxidase (HRP) from Jackson Immunoresearch Labs (Cat# 711-035-152 RRID: AB_10015282). Rabbit anti-goat conjugated to HRP from Molecular probes (Cat# R21459 RRID: AB_11180332). Rabbit anti-mouse conjugated to HRP from Sigma (Cat# A9044 RRID: AB_258431).

To immunolabel the Schwann cell, the presynaptic component of the NMJ and the target protein PDK1 we used: rabbit polyclonal anti-S100 antibody (Cat# Z0311 RRID: AB_10013383), from Dako. Rabbit monoclonal anti-syntaxin-6 antibody (Cat# C34B2 RRID: AB_10829116), from Cell Signaling Technology. PDK1 localization was performed with the same antibody used for Western blotting (Cat#sc-17765 RRID: AB_626657). The secondary antibodies used were donkey anti-mouse or anti-rabbit conjugated to Alexa Fluor 488 and Alexa Fluor 647 from Molecular Probes (Eugene, OR, USA; Cat# A21202 RRID: AB_141607; Cat# A31573 RRID: AB_2536183). Postsynaptic nicotinic acetylcholine receptors (AChRs) were detected with α-bungarotoxin (α-BTX) conjugated to Tetramethylrhodamine (TRITC) from Molecular Probes (Eugene, OR, USA; Cat# T1175 RRID: AB_2313931).

In Immunohistochemical and Western blot techniques, the absence of staining or bands when primary antibodies were omitted, served as a negative control. The appropriate blocking peptide was used to confirm the antibody specificity. Moreover, in double-staining protocols, one of the two primary antibodies were omitted to serve as a negative control.

### Presynaptic Electrical Stimulation of Muscles

Diaphragm muscle was dissected with the phrenic nerve into two hemidiaphragms and placed in oxygenated Ringer solution (in nM: NaCl 137, KCl 5, CaCl_2_ 2, MgSO_4_ 1, NaH_2_PO_4_ 1, NaHCO_3_ 12 and glucose 12.1 mM) continuously bubbled with 95% O_2_/5% CO_2_ at room temperature. One hemidiaphragm was used as the experimental condition and the other one as its control. Muscles were stimulated *ex vivo*, through their phrenic nerve at 1 Hz during 30 min by an A-M Systems 2100 isolated pulse generator (A-M System, Carlsborg, WA, USA). The main objective was to study independently the effect of synaptic transmission and the effect of the muscle cell contraction. To prevent muscle contraction, we used μ-conotoxin GIIIB (μ-CgTx-GIIIB, Alomone Labs Ltd, Israel; working solution 1.5 μM) that selectively inhibits sarcolemmal voltage-dependent sodium channels (VDSCs) without affecting synaptic ACh release (Favreau et al., 1999). Visible contractions of the diaphragm muscle indicated the successful nerve stimulation resulting in contraction. Table 1 show the experimental design of the treatments. The protocol of electrical stimulation applied was described in Besalduch et al. (2010); Hurtado et al. (2017) and Obis et al. (2015a). Briefly “In Experiment #1, synaptic activity effects were assessed by comparing presynaptically stimulated muscles blocked by μ-CgTx-GIIIB with non-stimulated muscles also incubated with μ-CgTx-GIIIB (referred to as the *Stimulation* condition in the figures). In Experiment #2, muscle contraction *per se* was assessed by comparing stimulated/contracting muscles to presynaptically stimulated muscles blocked by μ-CgTx-GIIIB (referred to as the *Contraction* condition in the figures). In Experiment #3, to assess the complete effect of synaptic activity with the resulting muscle contraction, we compared stimulated/contracting muscles with non-stimulated muscles, without incubate with μ-CgTx-GIIIB (referred to as the *Stimulation with Contraction* condition in the figures). At least five animals were used” (Hurtado et al., 2017).

**Table 1:** Summary of the electrical stimulation experiments.

Experiment	Control treatment		Treatment		Final outcome	
	**No stimulation, blocked contraction**		**Stimulation, blocked contraction**			
**#1 Presynaptic stimulation**	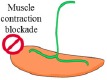	Hemidiaphragm extraction. μ-conotoxin GIIIB preincubation. Incubation in Ringer solution without stimulation.	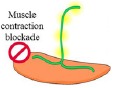	Hemidiaphragm extraction. μ-conotoxin GIIIB preincubation. Phrenic nerve stimulation with contraction blocked.	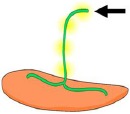	**Effect of presynaptic stimulation**.
	**Stimulation, blocked contraction**		**Stimulation, contraction**			
**#2 Contraction**	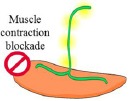	Hemidiaphragm extraction. μ-conotoxin GIIIB preincubation. Phrenic nerve stimulation with contraction blocked.	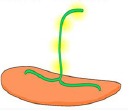	Hemidiaphragm extraction. Preincubation in Ringer solution. Phrenic nerve stimulation with contraction.	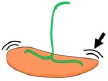	**Effect of muscle contraction**.	
	**No stimulation, not blocked contraction**		**Stimulation, contraction**			
**#3 Presynaptic stimulation with contraction**	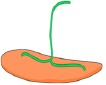	Hemidiaphragm extraction. Incubation in Ringer solution without stimulation.	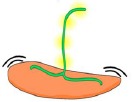	Hemidiaphragm extraction. Phrenic nerve stimulation with contraction.	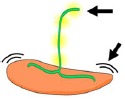	**Effect of presynaptic stimulation with contraction**.

*The Table has been published in the original article by Hurtado et al. (2017). The original article is an open access article distributed under the terms of the Creative Commons Attribution License (http://creativecommons.org/licenses/by/2.0), which permits unrestricted use, distribution, and reproduction in any medium, provided the original work is properly cited*.

### Western Blot

We obtained the samples as described in Hurtado et al. (2017). In brief, “diaphragm muscles were dissected, frozen in liquid nitrogen, and stored at −80°C before use. The muscles were homogenized using a high-speed homogenizer (overhead stirrer, VWR International, Clarksburg, MD, USA) in ice-cold lysis buffer (in mM: NaCl 150, Tris-HCl (pH 7.4) 50, EDTA 1, NaF 50, PMSF 1, sodium orthovanadate 1; NP-40 1%, Triton X-100 0.1% and protease inhibitor cocktail (1/100; Sigma-Aldrich, St. Louis, MO, USA). Insoluble material was removed by centrifugation at 1000 *g* for 10 min at 4°C. The supernatants were collected and centrifuged at 15,000 *g* for 20 min at 4°C. Finally, the resulting supernatants (total protein lysates) were collected. Protein concentrations were determined by using the Bio-Rad DC protein assay (Bio-Rad, Hercules, CA, USA). Experimental procedures were performed to determine the linear and quantitative dynamic range for each target protein and the appropriate dilutions of samples were used for accurate and normalized quantitation by means of densitometric analysis. To isolate the membrane and cytosolic fractions, diaphragm muscles were dissected and homogenized using a high-speed homogenizer in ice-cold lysis buffer without detergents (in mM: NaCl 150, Tris-HCl (pH 7.4) 50, EDTA 1, NaF 50, PMSF 1, sodium orthovanadate 1 and protease inhibitor cocktail (1/100). The homogenized samples were cleared at 1000 *g* for 15 min, and the resulting supernatant was further centrifuged at 130,000 g for 1 h. The supernatant was the cytosolic fraction and the pellet, the membrane fraction. The pellet was resuspended in lysis buffer (in mM: NaCl 150, Tris-HCl (pH 7.4) 50, EDTA 1, NaF 50, PMSF 1, sodium orthovanadate 1; NP-40 1%, Triton X-100 0.1% and protease inhibitor cocktail (1/100). Protein concentrations were determined in the same way as total protein lysates (see above). Validation of the purity of the subcellular fractionation was determined by examining the presence of fraction-specific housekeeping proteins like GAPDH for cytosol and Na/K-ATPase for membrane by Western blotting”.

Protein samples of 30 μg were separated through 8% SDS-polyacrylamide gels. After electrophoresis, the gels were transferred to a polyvinylidene difluoride (PVDF) membrane (Hybond™-P; Amersham, GE Healthcare) using Trans-Blot Turbo Transfer System (Bio-Rad, Hercules, CA, USA). For immunodetection, the membrane was blocked with Tris-buffered saline 0.1% Tween 20 (TBST) containing 5% (W/V) bovine serum albumin (BSA) for phosphorylated proteins and nonfat dry milk for non-phosphorylated proteins for an hour. Membranes were incubated with the primary antibody (specific for the interest protein) overnight at 4°C and then incubated with the corresponding secondary antibody linked to a HRP for 1 h. Finally, membranes were revealed with the Bio-Rad ECL kid and imaged with the ChemiDoc XRS+ Imaging System (Bio-Rad, Hercules, CA, USA).

Western Blot quantification between the experimental sample and the control was realized from the same blot image with the ImageJ software (ImageJ, RRID: SCR_003070). GAPDH and Na/K-ATPase proteins were used as loading controls, as well as total protein staining (Sypro Ruby protein blot stain, Invitrogen). The quantification values were normalized to: (1) the background and to (2) total protein quantification. Data are mean values ± SEM. Statistical significance of the difference between groups was evaluated under the Wilcoxon test or the Student’s *t*-test and the normality of the distributions was tested with the Shapiro-Wilk test. The criterion for statistical significance was *p* < 0.05 vs. the control (*) and at least five animals were evaluated in any condition.

### Immunohistochemistry and Confocal Microscopy

To localize PDK1 at the NMJ we performed immunohistochemistry in LAL muscle and diaphragm. Muscles were fixed for 30 min using 4% paraformaldehyde, then rinsed with phosphate buffer saline (PBS) and incubated in 0.1 M glycine in PBS. Then, muscles where incubated with goat serum overnight at 4°C, rinsed with PBS, and then incubated with 1% Triton X-100/4% BSA in PBS overnight at 4°C. Incubation with the primary antibodies, was done overnight at 4°C (anti PDK1; anti syntaxin to label the axon terminal; anti-S100 to label Schwann cells) and then rinsed with PBS. Finally, muscles were incubated in a mixture of appropriate secondary antibodies, overnight at 4°C. To detect AChRs we used α-BTX conjugated with TRITC. The appropriate negative controls were done in at least three muscles as described above. Moreover, there was not cross over between antibodies. For imaging, a laser-scanning confocal microscope (Nikon TE2000-E) was used and images were assembled using Adobe Photoshop software without modifying the contrast or brightness (Adobe Systems, San Jose, CA, USA; RRID: SCR_014199). Care was taken to the possible contamination of one channel by another. For negative controls imaging, the photomultiplier tube gains and black levels were not modified. At least 25 endplates per muscle were observed, and at least five muscles were studied.

## Results

### PDK1 in the Skeletal Muscle

**Figure 1:**
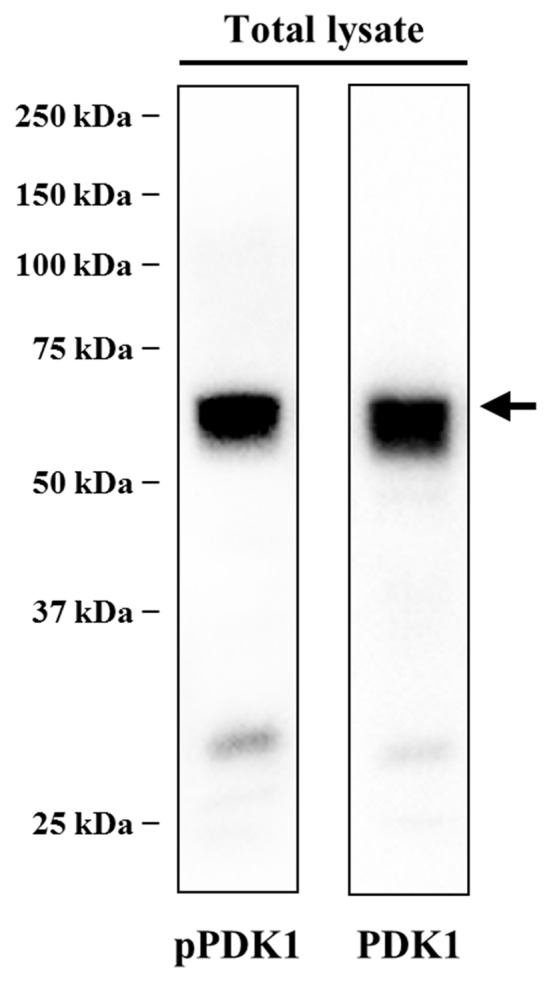
PDK1 is expressed in skeletal muscle in basal conditions. Determination of the specificity of anti-phosphorylated PDK1 (pPDK1) S241 antibody (CS-3061) and anti-PDK1 antibody (sc-17765) by immunoblotting. Thirty microgram of protein from total lysate were size fractionated by SDS-PAGE on 8% acrylamide minigels and transferred to polyvinylidene difluoride (PVDF) membranes. The antibodies used only recognized the corresponding protein, reacting with a band consistent with its predicted molecular weight. Western blot analysis revealed significant amounts of PDK1 and pPDK1 in the skeletal muscle in basal conditions. Abbreviations: PDK1, phosphoinositide-dependent kinase 1; pPDK1, phosphorylated phosphoinositide-dependent kinase 1.

Western blot analysis of PDK1 was carried out to determine its presence in the skeletal muscle. The anti-PDK1 antibody was raised against a peptide corresponding to the residues 229–556 of PDK1. This antibody revealed a major band of the predicted molecular weight (68 kDa), suggesting the monospecificity of the antibody (Figure 1). Phosphorylation of PDK1 was analyzed using an antibody raised against a peptide corresponding to the residues surrounding the Ser241 of human PDK1, a region identical to the rat PDK1 (Uniprot sequences O15530 and O55173, respectively). This antibody reacted with a unique band that is consistent with the PDK1 predicted molecular weight (Figure 1). Western blotting results revealed significant amounts of PDK1 and pPDK1 in the skeletal muscle in basal conditions (Figure 1). Subsequently, we sought to identify the cellular distribution of PDK1 at the NMJ by immunofluorescence.

### Localization of PDK1 in the Nerve Terminals of the NMJ

**Figure 2:**
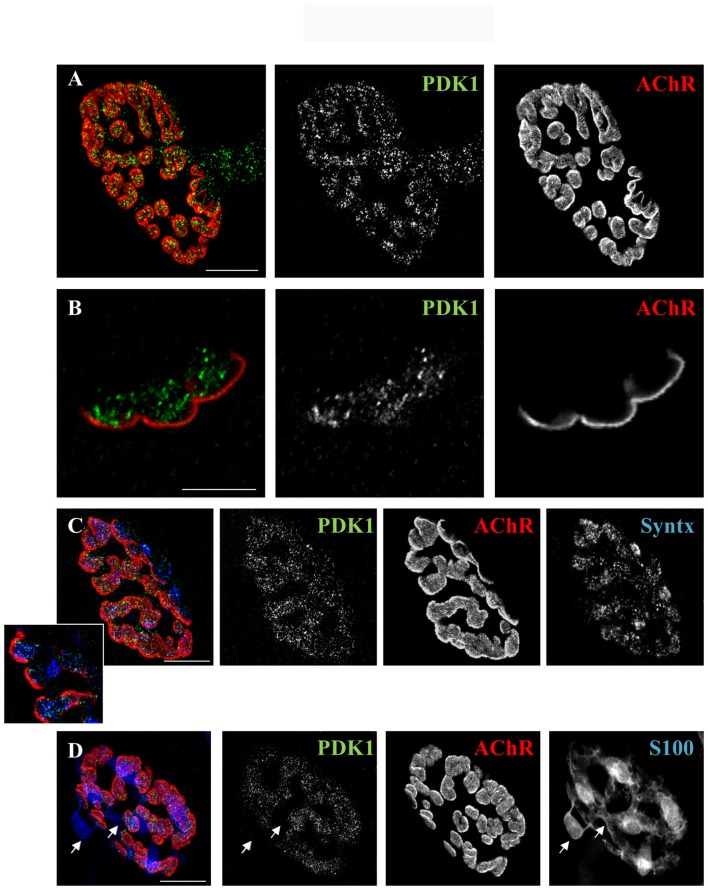
PDK1 is localized in the nerve terminals of the neuromuscular junction (NMJ). **(A,B)** Double staining labeled PDK1 (in green) and AChRs (fluorescent α-BTX in red). The images show PDK1-positive green immunolabeling concentrated at the presynaptic area over the red postsynaptic gutters. **(C)** Triple staining labeled PDK1 (in green), AChRs (in red) and nerve terminal (with anti-syntaxin antibody in blue, Syntx). The fine granulated label for PDK1 was well colocalized with syntaxin in the presynaptic nerve terminal position (over the AChRs-positive postsynaptic red gutters). **(D)** Triple staining labeled PDK1 (in green), AChRs (in red) and Schwann cell (with anti-S100 antibody in blue) showed that PDK1 was not colocalized with Schwann cell (see arrows). Thus, indicating that PDK1 is exclusively located at the presynaptic component of NMJ (*n* = 5; 25–30 endplates per muscle). The scale bars indicate 10 μm. Abbreviations: PDK1, phosphoinositide-dependent kinase 1; AChRs, Acetylcholine receptors; Syntx, syntaxin; S100, S100 protein; α-BTX, α-bungarotoxin.

The localization of PDK1 in the NMJ is essential to elucidate its function. Therefore, immunofluorescence coupled with confocal microscopy was carried out to stain PDK1 and the three cellular elements of the NMJ (*n* = 5; 25–30 endplates per muscle). Images in Figure 2 show intense immunoreactivity for PDK1 (in green) in the synaptic area identified with AChR labeling (in red). Figures 2A,B (cross-view confocal section) show a double labeled NMJ: AChRs (marked with fluorescently labeled α-BTX, in red) and PDK1 in green. These figures show PDK1-positive green immunolabeling concentrated at the presynaptic position over the red postsynaptic gutters, without immunoreactivity for muscle cells. Moreover, the pre-terminal axon was also PDK1-positive.

We also performed a triple staining in which we co-localized PDK1 (in green), muscle cell (AChR, in red), nerve terminal (labeled with syntaxin, in blue) and/or Schwann cells (labeled with S100, in blue; Figures 2C,D). The fine granulated label for PDK1 colocalized with syntaxin in the presynaptic nerve terminal position (over the AChRs-positive postsynaptic red gutters). The inset in Figure 2C shows a good colocalization between PDK1 and syntaxin indicating the presynaptic localization of PDK1 in the nerve terminal of the NMJ. Moreover, PDK1 was not colocalized with the Schwann cell (Figure 2D, see arrows). Altogether, these results indicate that PDK1 is exclusively located at the presynaptic component of NMJ.

### Total PDK1 Levels and Its Phosphorylation Are Unaltered after Synaptic Activity and Muscle Contraction

PDK1 is an upstream regulator of numerous protein kinases of the AGC kinase superfamily, including conventional PKC isoforms (Dutil et al., 1998). Previous results showed that pre- and postsynaptic neuromuscular activities regulate specifically cPKCβI protein levels (Besalduch et al., 2010) and its phosphorylation (Hurtado et al., 2017) in skeletal muscle total lysates. Therefore, our first objective was to determine whether synaptic activity and/or muscle contraction modulate PDK1 and its phosphorylation (pPDK1) in the skeletal muscle. In our *in vivo* experimental system, we can distinguish the effects of synaptic activity from those of muscle contraction. As described in Hurtado et al. (2017) “Synaptic activity includes the presynaptic events related with nerve stimulation (1 Hz, 30 min), synaptic transmission and endplate potential generation due to ACh signaling (referred to as the *Stimulation* condition in the figures). Muscle contraction includes membrane depolarization of the muscle fiber involving voltage-dependent sodium channels and the resulting myofiber contraction (referred to as the *Contraction* condition in the figures). Finally, presynaptic *Stimulation with Contraction* treatment includes the effects of synaptic activity and muscle contraction, showing complete neuromuscular activity”.

**Figure 3:**
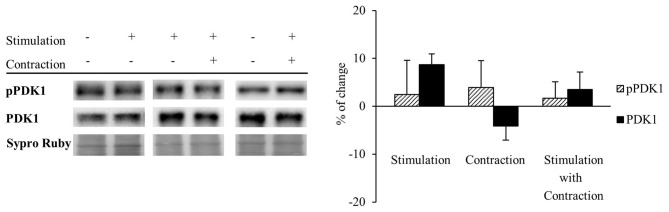
Total PDK1 levels and its phosphorylation are unaltered after synaptic activity and muscle contraction. PDK1 and pPDK1 in presynaptic stimulation treatment, Contraction treatment and Presynaptic stimulation with contraction treatment at 1 Hz stimulation for 30 min. Presynaptic stimulation has been simplified as *Stimulation*. Each column has been compared to its respective control (see Table 1). Results showed that PDK1 and pPDK1 levels, as well as pPDK1/PDK1 ratio, remained unaltered after nerve stimulation with and without muscle contraction. Data are mean percentage ± SEM, (Stimulation *n* = 7, Contraction *n* = 6 and Stimulation with Contraction *n* = 6). Abbreviations: PDK1, phosphoinositide-dependent kinase 1; pPDK1, phosphorylated phosphoinositide-dependent kinase 1.

We analyzed by Western blotting how activity affects the level of PDK1 and its phosphorylation (pPDK1) in total lysates. Results revealed that PDK1 and pPDK1 levels, as well as pPDK1/PDK1 ratio, remained unaltered after nerve stimulation with and without coincident muscle contraction (Stimulation *n* = 7, Contraction *n* = 6 and Stimulation with Contraction *n* = 6; Figure 3). This indicates that there is a stable pool of PDK1 at the NMJ catalytically competent during synaptic activity. However, although neuromuscular activity does not affect total PDK1 levels nor its phosphorylation, we recently determined that it induces the phosphorylation of its target cPKCβI. Therefore, PDK1 activation might be promoted by neuromuscular activity through PDK1 translocation to the plasma membrane.

### Synaptic Activity Increases Phosphorylated PDK1 and PCKβI in the Membrane Fraction of Skeletal Muscle

**Figure 4:**
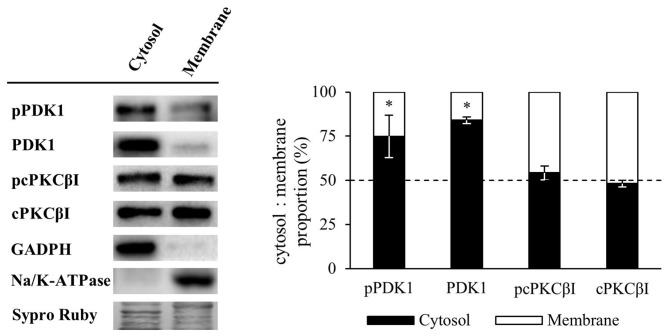
Membrane and cytosol distribution of PDK1 and cPKCβI in basal conditions. Western Blot analysis of the distribution of PDK1 and cPKCβI in membrane and cytosol fraction of skeletal muscle. Results showed that in basal conditions, pPDK1 and PDK1 were found predominantly in the cytosol fraction while pcPKCβI and cPKCβI were present similarly in both cytosol and membrane fractions. Moreover, glyceraldehyde 3-phosphate dehydrogenase (GAPDH) was found in the cytosol fraction and essentially undetectable in the membrane fraction. As expected, the membrane protein Na/K-ATPase was highly enriched in this cellular component, and undetectable in the cytosol fraction. Data are mean percentage ± SEM, *p < 0.05 (*n* = 5). Abbreviations: phosphoinositide-dependent kinase 1; pPDK1, phosphorylated phosphoinositide-dependent kinase 1; cPKCβI, conventional protein kinase C βI; pPKCβI, phosphorylated conventional protein kinase C βI.

Several lines of evidence show that PDK1 targeting to the plasma membrane is determinant for its activation (Yang et al., 2002a,b), leading to the phosphorylation of PKC, as it is also located in the plasma membrane. Thus, we proceeded to analyze how synaptic activity and/or muscle contraction modulate PDK1 and cPKCβI protein levels and their phosphorylation in the cytosol and membrane fractions. The purity of membrane and cytosol fractionation was confirmed by immunoblotting of Na/K-ATPase and GAPDH as specific protein markers. Results showed that the cytosolic protein GAPDH was in the cytosol fraction and essentially undetectable in the membrane fraction. As expected, the Na/K-ATPase was highly enriched in the membrane component, and undetectable in the cytosol fraction. Keranen et al. (1995) determined that only 50% of PKC species retain the PDK1-induced phosphate in their activation loop, being mature cPKCs quantitatively autophosphorylated at their turn-motif and hydrophobic loop. Therefore, to avoid the interference of dephosphorylation, we analyzed the phosphorylation of cPKCβI with an antibody against Thr642 turn-motif phosphorylation, which is the subsequent phosphorylation induced by PDK1 and it is required for kinase activity (Zhang et al., 1994). Our results showed that, in basal conditions, pPDK1 and PDK1 were found predominantly in the cytosol fraction (cytosol:membrane, pPDK1: 74.95:25.05% ± 12.02, *p* < 0.05; PDK1: 84.00:16.00% ± 1.84, *p* < 0.05; *n* = 5) while pcPKCβI and cPKCβI were present similarly in both cytosol and membrane fractions (cytosol:membrane, pcPKCβI: 54.21:45.79% ± 3.78, *p* > 0.05; cPKCβI: 48.28:51.72% ± 1.88; *p* > 0.05; *n* = 5; Figure 4).

**Figure 5:**
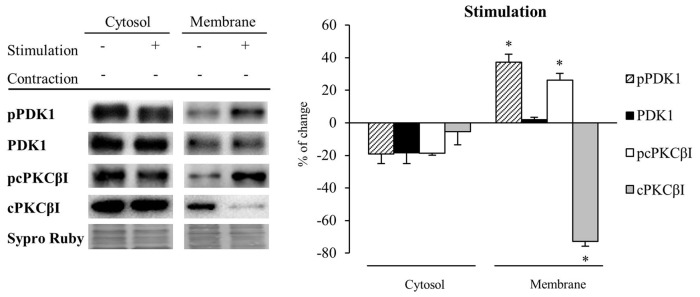
Synaptic activity increases pPDK1 and PCKβI in the membrane fraction of skeletal muscle. Western Blot of PDK1 and pPDK1 after isolation of membrane and cytosol fractions in presynaptic stimulation treatment at 1 Hz stimulation for 30 min. Presynaptic stimulation has been simplified as *Stimulation*. Each column has been compared to its respective control (see Table 1). Results showed that synaptic activity does not affect significantly the level of any protein in the cytosol. Therefore, the ratios pcPKCβI/cPKCβI and pPDK1/PDK1 remain the same. However, both pPDK1 and its substrate, pcPKCβI were significantly increased in the membrane fraction. Thus, both ratios pcPKCβI/cPKCβI and pPDK1/PDK1 were increased indicating that synaptic activity specifically enhances phosphorylation of PDK1 and cPKCβI. Data are mean percentage ± SEM, *p < 0.05 (*n* = 5). Abbreviations: phosphoinositide-dependent kinase 1; pPDK1, phosphorylated phosphoinositide-dependent kinase 1; cPKCβI, conventional protein kinase C βI; pPKCβI, phosphorylated conventional protein kinase C βI.

Next, we determined how synaptic activity without contraction affects the levels and the phosphorylation of PDK1 and cPKCβI in the cytosol and membrane fractions (*n* = 5; Figure 5). Results showed that synaptic activity does not affect significantly the level of any considered protein in the cytosol although the levels of PDK1, pPDK1 and pcPKCβI tended to decrease. Therefore, the ratios pPDK1/PDK1 and pcPKCβI/cPKCβI remained unchanged. However, synaptic activity significantly increased both pPDK1 and its substrate, pcPKCβI in the membrane (pPDK1: 37.31% ± 4.75, *p* < 0.05; pcPKCβI: 26.11% ± 4.15, *p* < 0.05). In addition, total protein levels of PDK1 were maintained and cPKCβI were significantly decreased (cPKCβI: −72.73% ± 3.12, *p* < 0.05; Figure 5). Thus, the increase in both pPDK1/PDK1 and pcPKCβI/cPKCβI ratios (35.88% ± 0.59, *p* < 0.05 and 362.95% ± 3.44, *p* < 0.05; respectively) indicate that synaptic activity enhances phosphorylation of PDK1 and cPKCβI. Together, these results show that presynaptic activity increases the levels of pPDK1 in the membrane fraction, a subcellular location known to be important for PDK1 function. Because this increase of pPDK1 coincides with a significant increase of pcPKCβI in the membrane fraction, this might indicate that synaptic activity increases PDK1 function to phosphorylate cPKCβI.

### Muscle Contraction Maintains PDK1 and pPDK1 Levels but Increases cPKCβI and pcPKCβI Levels in the Membrane Fraction of Skeletal Muscle

**Figure 6:**
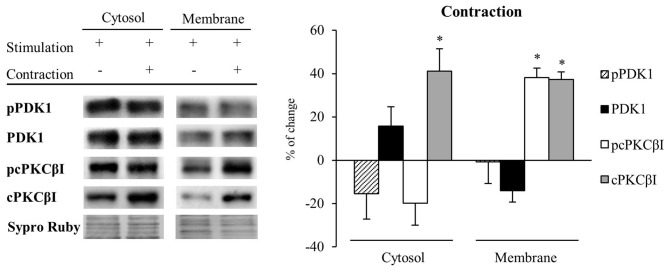
Muscle contraction maintains PDK1 and pPDK1 levels but increase cPKCβI and pcPKCβI levels in the membrane fraction of skeletal muscle. PDK1 and pPDK1 after isolation of membrane and cytosol fractions in Contraction treatment at 1 Hz stimulation for 30 min. Each column has been compared to its respective control (see Table 1). We observed that muscle contraction increased cPKCβI protein levels in the cytosolic fraction but pPDK1, PDK1 and pcPKCβI levels remained the same. Thus, the ratio pPDK1/PDK1 was maintained while pcPKCβI/cPKCβI decreased due to the increase of the total protein. Moreover, in the plasma membrane, pPDK1 and PDK1 were also not modified but pcPKCβI and cPKCβI were significantly increased. Thus, the ratio pPDK1/PDK1 and pcPKCβI/cPKCβI in the membrane fractions remained the same. Data are mean percentage ± SEM, *p < 0.05 (*n* = 5). Abbreviations: phosphoinositide-dependent kinase 1; pPDK1, phosphorylated phosphoinositide-dependent kinase 1; cPKCβI, conventional protein kinase C βI; pPKCβI, phosphorylated conventional protein kinase C βI.

Because muscle activity *per se* has a critical role to enhance presynaptic cPKCβI (Besalduch et al., 2010; Hurtado et al., 2017), we analyzed the role of muscle contraction over PDK1 and cPKCβI protein levels and their phosphorylation in the cytosolic and membrane fraction (*n* = 5; Figure 6). We observed that muscle contraction increased cPKCβI protein levels in the cytosolic fraction (cPKCβI: 41.22% ± 10.29, *p* < 0.05) without altering pPDK1, PDK1 and pcPKCβI levels. Thus, the ratio pPDK1/PDK1 was maintained while pcPKCβI/cPKCβI decreased due to the increase of the total cPKCβI levels (−43.09% ± 1.62, *p* < 0.05). So even though PKCβI is increased, muscle contraction does not promote its phosphorylation in the cytosol. Regarding the membrane fraction, pPDK1 and PDK1 levels did not change after contraction but both pcPKCβI and cPKCβI were significantly increased (pcPKCβI: 38.19% ± 4.35 *p* < 0.05; cPKCβI: 37.23% ± 3.50, *p* < 0.05). Thus, the ratio pcPKCβI/cPKCβI in the membrane fraction remained the same indicating that muscle contraction enhances phosphorylation of cPKCs due to an increase of total protein PKC protein level.

These results together suggest that muscle contraction might induce the synthesis of cPKCβI, increasing total protein levels in both cytosol and membrane fractions. The increased amount of pcPKCβI in the membrane might be explained by an increase of total PKC protein level, as PDK1 activity is maintained (see Figure 6).

To reinforce the previous results, we assessed the complete neuromuscular activity (synaptic activity with muscle contraction; *n* = 5). In the membrane fraction, pPDK1 was increased (achieved by synaptic activity; pPDK1: 40.77% ± 9.27, *p* < 0.05) but without altered PDK1 protein levels (PDK1: 11.37% ± 1.58, *p* > 0.05). Moreover, total cPKCβI and pcPKCβI levels were also increased in the membrane fraction due to muscle contraction (cPKCβI: 31.88% ± 8.59, *p* < 0.05; pcPKCβI: 30.42% ± 8.19, *p* < 0.05).

Altogether, these results suggest that synaptic activity induces the phosphorylation of cPKCβI through the translocation of pPDK1 to the membrane. Furthermore, contraction increases the synthesis of cPKCβI and consequently the amount of pcPKCβI even maintaining PDK activity.

## Discussion

PDK1 is a crucial Ser/Thr kinase which activates as many as 23 protein kinases of the AGC family, including PKC, by phosphorylating their T-loop sites (Toker, 2003; Mora et al., 2004; Bayascas, 2010; Pearce et al., 2010). Although the importance of PDK1 in PKC signaling has been well characterized (Chou et al., 1998; Dutil et al., 1998; Le Good et al., 1998; Balendran et al., 2000), its synaptic localization and function in the nervous system has not been fully determined. Thus, in this study, we localized PDK1 at the neuromuscular synapse and we investigated the hypothesis that synaptic activity and muscle contraction regulates PDK1 and cPKCβI phosphorylation in the membrane fraction. Our results support that PDK1 is localized in the nerve terminals of the NMJ. Moreover, synaptic activity increases pPDK1 levels in the membrane. Because the increase of pPDK1 coincides with a significant increase of pcPKCβI in the membrane fraction, this might indicate that synaptic activity increases PDK1 function to phosphorylate cPKCβI. Furthermore, when contraction is present, the total amount of cPKCβI is increased in both cytosol and membrane fraction, suggesting an activation of its synthesis.

### Synaptic Activity Increases Phosphorylated PDK1 and pcPKCβI in the Membrane Fraction of the Skeletal Muscle

In the skeletal muscle, PDK1 is mainly present in the cytosolic fraction in basal conditions and the confocal microscopy shows that it is only expressed in the nerve terminal of the rat NMJ. Consistent with that, PDK1 has been located also in the nerve terminals at the *Drosophila* NMJ (Cheng et al., 2011). It is surprising that while several PKC isoforms are located in the different cells of the rat NMJ (Perkins et al., 2001; Besalduch et al., 2010, 2013; Lanuza et al., 2014; Obis et al., 2015a), PDK1 is located exclusively in the nerve terminal. This fact may be related with a specific role of this protein in priming presynaptic kinases (such nPKCε and cPKCβI) selectively involved in the rapid and complex exocytotic process of transmitter release. Due to its presynaptic location, PDK1 activation could be susceptible to synaptic activity influence. Different studies have shown that PDK1 is constitutively phosphorylated on at least five serine residues (S25, S241, S393, S396 and S410; Casamayor et al., 1999). However, other studies suggest that signaling pathways activated by insulin-like growth factor 1 (IGF-1) can further increase the degree of PDK1 phosphorylation on these sites (Scheid et al., 2005). Our results suggest that PDK1 is constitutively phosphorylated in the S241 site after synaptic activity and muscle contraction in total skeletal muscle lysates. However, we demonstrated that the subcellular localization of pPDK1 is inducible by synaptic activity. Activity is able to translocate pPDK1 to the plasma membrane where PDK1 is in the optimal situation to interact with and phosphorylate its substrates (Chou et al., 1998; Dutil et al., 1998; Le Good et al., 1998; Balendran et al., 2000; Yang et al., 2002a,b). It should be noted that pPDK1 is slightly, but not significantly, reduced in the cytosol fraction and it may be because PDK1 is mainly present in the cytosol fraction. Thus, small decreases in their protein levels might not be significantly appreciated, but enough to detect a significant increase in the membrane fraction. PI3-kinase (PI3K) activity recruits PDK1 to membranes, leading to phosphorylation of downstream substrates (Alessi et al., 1997). Here we show that this recruitment to membrane is promoted by the synaptic activity at the NMJ and this mechanism may be Ca^2+^ dependent. PDK1 with its PH domain binds to either PIP3 or PIP2 and is translocated to the plasma membrane. Evidence show that PDK1 does not have any domain that directly interacts with calcium. However, recent evidence shows that in central nerve terminals an increase of intracellular calcium promotes PI3K activity by an unknown calcium sensor (Nicholson-Fish et al., 2016). Therefore, calcium influx may increase PIP3 production (by enhancing PI3K) which, in turn, could promote PDK1 translocation to the membrane. It has been evidenced that PDK1 is the upstream kinase which directly phosphorylates the activation loop of PKC isoforms (Dutil and Newton, 2000). Although PDK1 is constitutively active (Casamayor et al., 1999) the translocation to the membrane induced by synaptic activity may provide an important mechanism for prolonged activation of PKCs.

PKC family has emerged as essential for the control of aspects of higher-level signal organization. It is a multigene family of Ser/Thr kinases that comprises ~2% of the human kinome. In the nervous system, synaptic transmission (Dempsey et al., 2000; Lanuza et al., 2007; Tomàs et al., 2014) is decisively modulated by the involvement of several PKC isoforms differently localized and regulated (Hilgenberg and Miles, 1995; Lanuza et al., 2000; Perkins et al., 2001; Li et al., 2004; Besalduch et al., 2010, 2013; Obis et al., 2015a,b). For instance, the novel nPKCθ has several roles which include the neuromuscular system development (Li et al., 2004; Lanuza et al., 2006, 2010; Besalduch et al., 2011) and differentiation and homeostasis of the skeletal muscle (Tokugawa et al., 2009; Madaro et al., 2011, 2012). nPKCθ may regulate excitability and muscle contraction through the modulation of chloride channel activity (Camerino et al., 2014). In addition, the novel nPKCε coupling is clearly involved to maintain or potentiate ACh release in the NMJ (Obis et al., 2015b). Interestingly, conventional cPKCβI is exclusively located in the presynaptic component, is modulated by both synaptic activity and muscle contraction and, in turn, is directly involved in transmitter release in the NMJ (Besalduch et al., 2010; Hurtado et al., 2017). It is interesting to note that PDK1, as well as cPKCβI, is exclusively located in the nerve terminal at the NMJ.

To become competent and able to respond to second messengers, PKCs undergo a previous process of maturation (Parekh et al., 2000; Newton, 2003) and its activation requires translocation of the enzyme to membrane (Kraft et al., 1982). Conventional cPKC maturation involves three phosphorylation steps at specific sites, the first of which is mediated by PDK1 in the catalytic domain activation loop. In contrast, the two carboxy-terminal phosphorylations in the turn and hydrophobic motifs have been shown to undergo autophosphorylation events subsequent to the PDK1 mediated phosphorylation (Cazaubon and Parker, 1993; Keranen et al., 1995; Dutil et al., 1998). Membrane location confers to PKC a permissive change that promotes activation loop phosphorylation by PDK1. Mature cPKCs, are released into the cytosol and kept in an inactive conformation ready to be activated (Oancea and Meyer, 1998; Violin et al., 2003; Griner and Kazanietz, 2007). However, recent findings show that PKCs can also exist in non/hypophosphorylated forms, with cellular stimulation resulting in inducible phosphorylation and activation (Zhou et al., 2003; Wang et al., 2007; Osto et al., 2008). Obis et al. (2015a) described that synaptic activity modulates phosphorylation of nPKCε at the NMJ. Moreover, presynaptic cPKCβI phosphorylation is enhanced by synaptic activity and muscle contraction (Hurtado et al., 2017). Here, our results showed that phosphorylation of cPKCβI is inducible by synaptic activity and specifically increased in the plasma membrane. Thus, at the membrane fraction, the significant increase of pPDK1 described above, coincides with a significant increase of pcPKCβI. Because PDK1 directly interacts with cPKCβI, among other PKC isoforms, through the kinase domain of the enzyme (Dutil et al., 1998; Le Good et al., 1998), this might indicate that synaptic activity increases PDK1 function to phosphorylate cPKCβI allowing for its substrate binding, phosphorylation and the activation of downstream signaling effectors. The increase of pcPKCβI in the membrane after synaptic activity is accompanied by a significant decrease of the total cPKCβI indicating the described downregulation of the PKC after activation. This result was also previously demonstrated (Hurtado et al., 2017) and here we specifically found that is in the membrane where the downregulation occurs. Furthermore, total but not phosphorylated cPKCβI protein levels depends on synaptic activity-induced BDNF/TrkB signaling at the NMJ (Hurtado et al., 2017) indicating that PDK1 activity phosphorylating cPKCβI would be not modulated by the BDNF/TrkB signaling pathway.

### Muscle Contraction Maintains PDK1 and pPDK1 Levels but Increases cPKCβI and pcPKCβI Levels in the Membrane Fraction of Skeletal Muscle

PDK1 has been related with cell contraction and cell migration. Some studies suggest that the kinase activity of PDK1 was not required for the regulation of cortical subplasmalemic actin or cell contraction (Pinner and Sahai, 2008); this contrasts with previous reports suggesting that PDK1 regulates actin organization through PKB/Akt, PAK or integrinβ3 (Lim et al., 2004; Weber et al., 2004; Xie et al., 2006; Primo et al., 2007). However, whether PDK1 activity is related with muscle contraction *in vivo* in the skeletal muscle is still unknown. Here we show that muscle contraction does not modify pPDK1 and PDK1 levels either in the cytosol or the membrane fraction, suggesting that its activity is mainly determined only by presynaptic activity. This result is consistent with the exclusive location of the PDK1 that we found, in the nerve terminal of the NMJ and suggests that it is not retrogradely regulated by the muscular activity.

However, PKC isoforms are differently regulated in the skeletal muscle (Hilgenberg and Miles, 1995; Lanuza et al., 2000; Perkins et al., 2001; Li et al., 2004; Besalduch et al., 2010, 2013; Obis et al., 2015a,b). Especially, our results show that conventional cPKCβI is modulated by muscle contraction in both cytosol and membrane fractions, as previously demonstrated (Besalduch et al., 2010; Hurtado et al., 2017). Specifically, we observed that muscle contraction increased cPKCβI protein levels in the cytosolic fraction suggesting that it is thus promoting its synthesis or alternatively decreasing its degradation. In addition, in the plasma membrane both pcPKCβI and cPKCβI were significantly increased suggesting that PKC synthesis, its translocation to the membrane and its phosphorylation are enhanced after muscle contraction. Thus, these results indicate that muscle contraction induces the synthesis of cPKCβI, increasing total protein levels in both cytosol and membrane fractions. Furthermore, it has been shown that presynaptic cPKCβI levels are enhanced by muscle contraction through the BDNF/TrkB signaling suggesting a retrograde regulation of this isoform (Hurtado et al., 2017). However, even though pPDK1 activity is maintained, pcPKCβI is enhanced due to the increased amount of total cPKCβI caused by muscle contraction.

**Figure 7:**
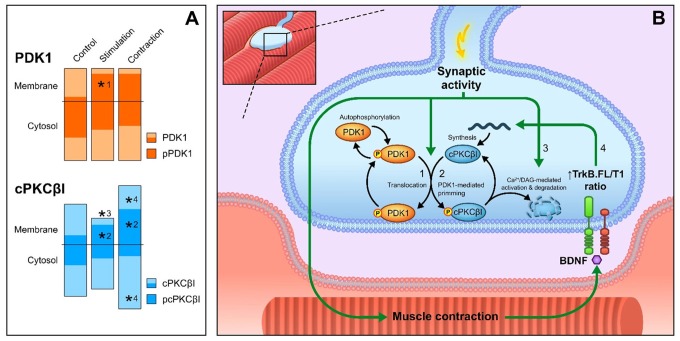
Overview of PDK1 function and cPKCβI phosphorylation at the NMJ. **(A)** Summary of the results of this work. Columns represent the total protein levels of PDK1 (in orange), pPDK1 (in dark orange), cPKCβI (in blue) and pcPKCβI (in dark blue) in the membrane and cytosol compartments (indicated with a horizontal line) *p < 0.05. **(B)** Proposed model of the action of PDK1 on cPKCβI phosphorylation at the NMJ. Total PDK1 protein and phosphorylation levels are maintained throughout all conditions. (#1) Synaptic activity induces the translocation of pPDK1 to the membrane. Consistent with the increased pPDK1 in the membrane, synaptic activity also (#2) increases the phosphorylation of cPKCβI in the same compartment. Once catalytically competent, pcPKCβI activation through synaptic activity (#3) reduces the total amount of cPKCβI in the membrane due to an increase in its activation-induced degradation. Muscle contraction (#4) increases the total amount of cPKCβI in both cytosol and membrane, suggesting an activation of its synthesis. In previous work, we determined that this enhancement is retrogradely regulated through BDNF/TrkB signaling (Hurtado et al., 2017). After muscle contraction, PDK1 activity remains unaltered and, therefore, pcPKCβI levels increase in concordance with total cPKCβI (#2). Abbreviations: phosphoinositide-dependent kinase 1; pPDK1, phosphorylated phosphoinositide-dependent kinase 1; cPKCβI, conventional protein kinase C βI; pPKCβI, phosphorylated conventional protein kinase C βI; BDNF, brain-derived neurotrophic factor; TrkB.FL, tropomyosin-related kinase B full-length, TrkB.T1, tropomyosin-related kinase B truncated isoform 1.

Figure 7A summarizes our results. Thus, this study demonstrates that PDK1 is exclusively located in the nerve terminal of the NMJ and that synaptic activity enhances the location of its phosphorylated form in the membrane (Figure 7-#1), the optimal place to be active. This increment of the levels of pPDK1 in the membrane coincides with increases of its substrate pcPKCβI in the membrane (Figure 7-#2), suggesting that synaptic activity increases PDK1 function to phosphorylate cPKCβI. Synaptic activity reduces the total amount of cPKCβI in the membrane due to an increase in its activation-induced degradation (Figure 7-#3). The resulting muscle contraction may play a retrograde control over presynaptic cPKCβI to activate its synthesis (or alternatively decreasing its degradation), thus increasing the amount of cPKCβI (Figure 7-#4, Hurtado et al., 2017). This might explain the increase in pcPKCβI (Figure 7-#2). The diagram in Figure 7B shows this proposed mechanism of the action of PDK1 on cPKCβI phosphorylation at the NMJ. Importantly, pcPKCβI has a critical role in regulating transmitter release (Hurtado et al., 2017). Thus, both pre- and postsynaptic activities are needed to modulate PDK1 and cPKCβI function, ensuring an accurate neurotransmission process.

## Ethics Statement

The animals were cared for in accordance with the guidelines of the European Community Council Directive of 24 November 1986 (86/609/EEC) for the humane treatment of laboratory animals. All the procedures realized were reviewed and approved by the Animal Research Committee of the Universitat Rovira i Virgili (Reference number: 0289).

## Author Contributions

EH: data collection, quantitative analysis, literature search, data interpretation, statistics; VC: data collection, literature search, data interpretation, design graphic abstract; LJ, LN, AS and MT: data collection; JT, MAL and NG: conception and design, literature search, data interpretation, manuscript preparation.

## Conflict of Interest Statement

The authors declare that the research was conducted in the absence of any commercial or financial relationships that could be construed as a potential conflict of interest.

## Abbreviations

ACh: acetylcholine
AChRs: acetylcholine receptors
α-BTX: α-bungarotoxin
BDNF: Brain-derived neurotrophic factor
BSA: bovine serum albumin
DAG: diacylglycerol
GAPDH: glyceraldehyde 3-phosphatedehydrogenase
HRP: horseradish peroxidase
LAL: levator auris longus
μ-CgTx-GIIIB: μ-conotoxin GIIIB; neurotrophic factor
NMJ: neuromuscular junction
PBS: phosphate buffer saline
PDK1: phosphoinositide-dependent kinase 1
PKC: Protein kinase C
PVDF: polyvinylidene difluoride
Ser: serine
Thr: threonine
TRITC: Tetramethylrhodamine
TrkB: tyrosine receptor kinase B
TSBT: Tween 20.
